# GP specialty trainees’ knowledge and values towards physical activity: a national survey of Scottish trainees

**DOI:** 10.3399/BJGPO.2023.0051

**Published:** 2024-01-10

**Authors:** Callum Leese, Robert H Mann, Emma J Cockcroft, Kirstin Abraham

**Affiliations:** 1 School of Medicine, University of Dundee, Dundee, UK; 2 Department of Public Health and Sport Sciences, University of Exeter Medical School, Exeter, United Kingdom; 3 Department of Health and Community Sciences, University of Exeter Medical School, Exeter, UK

**Keywords:** education, physical activity, exercise, primary health care

## Abstract

**Background:**

Despite the known benefits of physical activity (PA), one-third of adults in the UK fail to meet recommended levels of PA. PA promotion in primary care has been shown to be effective at improving PA in patients but implementation of PA promotion by GPs remains poor. Research has shown a need to improve PA education in undergraduate medical education, but, to the authors’ knowledge, no review of postgraduate medical education has been performed.

**Aim:**

To assess the knowledge and values of general practice specialist trainees (GPSTs) towards PA promotion in Scotland.

**Design & setting:**

Cross-sectional survey distributed to GPSTs in Scotland.

**Method:**

A mixed-methods cross-sectional survey, informed by previous research, was developed and distributed to all (*n* = 1205) GPSTs in Scotland in December 2022. Descriptive statistics were used to analyse quantitative data. A content analysis of free-text responses was also performed.

**Results:**

A total of 168 GPSTs responded, representing 13.9% of all GPSTs in Scotland. Of responders, 93.5% reported no previous experience in the subject of sports and exercise science and medicine. Overall, 38.9% of responders stated they were unaware of the current UK PA guidelines, with 33.9% unable to correctly identify the UK PA guidelines when presented with multiple choice options. In addition, 83.3% felt they had been inadequately trained to deliver PA advice during their medical training.

**Conclusion:**

This study highlights a lack of knowledge, confidence, and education in PA promotion in GPSTs in Scotland. Given the importance of primary prevention, this urgently needs to be addressed.

## How this fits in

Promotion of physical activity (PA) in primary care has been shown to increase patients' PA. This novel work highlights for the first time that future GPs do not feel adequately trained in PA promotion. This manifests in a lack of knowledge and confidence in discussing PA with patients. This urgently needs to be addressed, given the burden of physical inactivity on health care and society as a whole.

## Introduction

Research has demonstrated that regular PA results in extensive physical and mental health benefits.^1^ In 2019, the Chief Medical Officer for the UK introduced updated PA guidelines, building on previous iterations. These guidelines recommend that adults should aim to accumulate at least 150 minutes of moderate-intensity aerobic exercise per week, including at least two sessions weekly aimed at muscle strength and balance.^2^ These recommendations align with the latest World Health Organization PA guidance released in 2020.^3^ Despite this, one-third of adults in the UK fail to meet the 2019 Chief Medical Officer’s PA guidelines.^4^ This is important as physical inactivity results in huge detrimental implications on an already stretched health service.^4^ For example, the Department for Health and Social Care of England and Wales has reported that a lack of PA is associated with one in six deaths in the UK and costs the NHS £0.9 billion annually (and £7.2 billion to the UK economy).^4^

Lifestyle interventions delivered via primary care (for example, brief interventions promoting walking) have been shown to be effective at initiating behavioural change and reducing the risk of disease progression.^5^ A recent systematic review by Kettle and colleagues^6^ showed that PA interventions delivered in primary care are effective at increasing PA in patients. Furthermore, research has shown PA promotion interventions within primary care to be cost-effective,^7^ with a shifting emphasis within healthcare settings from treatment to cure.^8^

Despite primary care being a key point of influence for PA behaviours, a 2017 Royal College of General Practitioners (RCGP) and Public Health England (PHE) survey evidenced poor implementation of PA promotion by GPs.^9^ A frequently cited reason for this is a lack of knowledge among health professionals about the benefits of PA and how to appropriately promote it.^10–18^ In a nationwide survey of GPs in England, only 20% of responders (*n* = 203) were broadly familiar with the UK PA guidelines, and 55% (*n* = 557) reported they had not undertaken any training regarding PA counselling or advice.^9^ A failure of medical school education in the UK was suggested by Weiler and colleagues,^19^ whereby their assessment of medical school curricula demonstrated that only 56% of medical schools taught the Chief Medical Officer’s guidelines on PA to medical students, with a mean time spent teaching the benefits of PA of 4.2 hours. These findings were supported by a study of Scottish final-year undergraduate medical students, which revealed that only 40% were aware of current PA guidelines.^20^ Inadequate education in PA is not isolated to UK medical schools, with concerns raised in the US and Australia.^21,22^ It is also not a new problem, with inadequate PA education first highlighted in 2000,^23^ with an editorial published in the *British Journal of Sports Medicine* in 2023 calling for the issue to be re-addressed in response to the lack of change.^24^ This reported lack of change is supported by a recent survey of undergraduate medical students in London.^25^

Given the known benefits of PA and the effectiveness (including cost-effectiveness) of PA promotion in primary care, training GPs of the future is crucial for enacting positive change. Despite research having been done within the context of undergraduate medical education, there is an absence of research reviewing postgraduate medical education. This is especially the case within primary care, which is often viewed as a cornerstone of public health messaging and disease prevention owing to its unconditionality, longevity, and person-centred approach, while remaining the most widely accessed part of health care (for example, >300 million GP consultations yearly in the UK).^26,27^ Within the UK, GP training is a 3-year programme combining primary and secondary care exposure that is regulated by the RCGP and General Medical Council (GMC), combining exposure-based and outcome-based learning. In Scotland, it is a combination of 18 months of primary care and 18 months in secondary care (3 × 6-month blocks of differing specialties).

PA is a crucial part of disease prevention, management, and treatment,^1^ with adequate education of GPs essential to ensure this is delivered appropriately and effectively. This study therefore aimed to address whether GPs are adequately educated by assessing knowledge and values towards PA promotion in general practice specialist trainees (GPSTs).

## Method

A mixed-methods cross-sectional survey was developed using an online survey tool (Jisc). The questions were compiled by an advisory panel including NHS Education Scotland (NES) representatives (*n* = 2), academic researchers (*n* = 2), and a GP with a special interest in PA (*n* = 1). The study design was informed by research previously performed by Dunlop and Murray^20^ within an undergraduate medical setting. The survey aimed to assess all previous training related to PA (five questions), level of knowledge regarding the UK PA guidelines (two questions), and perceived confidence (three questions) through a range of single best answer, five-point Likert scale, and free-text questions. The survey can be found in Supplementary Information S1. The survey was disseminated to all GPSTs in Scotland via email from the NES database on 24 November 2022. A reminder email was sent on 9 December 2022, with the survey closing on 24 December 2022. Data analysis was performed using SPSS Amos (version 28.0.0.0 [190]).

Descriptive statistics were used to present quantitative data. As the study only aimed to describe GP trainees’ experience of education, knowledge of PA, and confidence in PA promotion, inferential statistics were not used. Free-text responses were independently reviewed by two authors (CL and KA) to identify themes using NVivo software (version 1.5) in a conventional content analysis, as described by Hsieh and Shannon.^28^ Following this, discussion between all authors of independent subthemes resulted in the creation of three themes identified in this study.

## Results

A total of 168 GPSTs responded, representing 13.9% of all GPSTs in Scotland (*n* = 1205) at the time the survey was distributed. Of the GPST cohort in Scotland (*n* = 1205), 468 were in GPST3 (response rate of 18.4%, *n* = 86), 405 were in GPST2 (response rate of 11.9%, *n* = 48), and 332 were in GPST1 (response rate of 10.2%, *n* = 34). More than half of the sample (*n* = 86, 51.2%) were in their final year of the 3-year programme (GPST3s), while 28.6% (*n* = 48) were in the second year (GPST2s), and 20.2% (*n* = 34) were in the first year (GPST1s), respectively. The mean time to complete the survey was 2 minutes and 20 seconds.

Of all responders, 93.5% (*n* = 157) reported no previous experience in the subject of sports and exercise science and medicine. Experience reported by 11 participants included undergraduate, intercalated, and master’s degrees in sports and exercise science and medicine, in addition to one responder with a personal training qualification.

Overall, 38.9% (*n* = 65) of responders stated they were not aware of the current UK PA guidelines, while 33.9% (*n* = 57) were unable to correctly identify the UK PA guidelines for adults. Of the GPSTs who stated they were aware of the guidelines, 83.3% (*n* = 38/46) were able to correctly identify them. Conversely, of those GPSTs who stated they were not aware of the guidelines, 39.7% (*n* = 25) correctly identified them. Knowledge of the UK PA guidelines increased with training, with 47.1% of GPST1s (*n* = 16) able to correctly identify the guidelines compared with 70.9% (*n* = 61) of GPST3s.

GPSTs acknowledged the importance of PA in preventing disease (100% agreement) and treating disease (97.0% agreement), but 51.8% (*n* = 87) of responders did not feel confident in advising patients regarding PA (see Supplementary Figure S1). Confidence in advising patients on PA did not improve as GPSTs progressed through training (47.1%, *n* = 16 of GPST1s felt confident compared with 47.6%, *n* = 41 of GPST3s).

Regarding their own training in PA promotion, 83.3% (*n* = 140) of responders felt it had been inadequate (see Supplementary Figure S2). Only 21.4% (*n* = 36) and 23.2% (*n* = 39) of responders felt that their undergraduate or postgraduate medical curriculum provided good teaching to enable them to advise patients, respectively.

Free-text responses (*n* = 39) revealed the following three main themes: 1) there was a lack of teaching in PA in both the undergraduate and postgraduate curriculum; 2) the available teaching tended to focus on the benefits of PA but lacked teaching on the deliverance of PA promotion; and 3) the opportunities available were often optional and/or independent of GP training (see Supplementary Table S1).

## Discussion

### Summary

The majority of GPSTs in Scotland (93.5%) had no previous experience in the subject of sport and exercise science and medicine, with 38.9% not aware of the UK PA guidelines. However, GPSTs agreed that PA was important in disease prevention (100%) and disease treatment (97.0%). Despite this, 51.8% did not feel confident in advising patients on PA, with 83.3% feeling that they had been inadequately trained to provide PA advice and promotion.

### Strengths and limitations

To the best of the authors' knowledge, this is the first study of GP trainees in the UK regarding attitudes to PA. It does, however, have several limitations. The analysis was performed on a small number of responders (*n* = 168), with a response rate of 13.9% of all GPSTs in Scotland. This compares with the response rate in a survey of GPs in England by Chatterjee and colleagues of 12.1%.^9^ This response rate may have been influenced by unprecedented pressures on health professionals at the time of the survey. As with all survey-based research, response rate may have an impact via responder bias. This may include more responders who have a pre-existing interest in PA. Participants were only from Scotland, and so the findings do not represent all postgraduate primary care deaneries in the UK. A responder bias was noted towards GPST3s, with 18.4% of GPST3 responding compared with 10.2% of GPST1s and 11.9% of GPST2s. This, however, does offer more insight into the postgraduate curriculum across Scotland as GPST3s near completion of their training.

### Comparison with existing literature

Despite the majority of GPSTs supporting the benefits of PA, 38.9% were not aware of the current UK PA guidelines. Confidence in giving PA advice was low, with 51.8% of GPSTs not feeling confident in the provision of PA advice. Research on PA promotion within fully qualified GPs has shown only 35.1% reported being at least ‘somewhat familiar’ with the PA guidelines, but 74.1% felt confident raising the topic of PA with their patients.^29^ Given the role primary care plays in promoting PA,^5–7^ being unfamiliar with the guidelines is concerning and needs to be addressed.

Within primary care it has been established that a lack of education on PA is seen as the main barrier to its promotion.^29^ Despite this issue being repeatedly raised over the past decade (particularly regarding the undergraduate curriculum),^19–22^ this research identifies that lack of education is ongoing; only 21.4% of responders felt their undergraduate teaching and 23.5% felt their postgraduate teaching on PA had been good. This was lower than figures reported in final-year medical students in 2014, with 52% feeling they have been adequately trained to give PA advice to the general population.^20^ Similarly, among fully qualified GPs, a 2016 survey revealed 55% had not undertaken any training in PA promotion.

The broad nature of barriers in postgraduate education are consistent with undergraduate medical education, including a lack of curriculum space, time, and qualified educators.^19,23,30^ However, medical education needs to adapt. A knowledge and experience of behavioural change and motivational interview techniques within primary care are increasingly important for PA promotion. This research suggests that the postgraduate curriculum in primary care needs to provide training in these techniques. A novel approach by Maini and colleagues^31^ found that using medical students as health coaches was an effective way of improving medical student self-efficacy and communication skills. This provides a possible template, both for undergraduate and postgraduate medical education. Within primary care, the RCGP has attempted to address the need for education by launching the Active Practice Charter in 2019^32^ and, more recently, the parkrun practice initiative.^33^ Despite this, GP training offers a unique platform for education at an early career stage with a captive audience.

### Implications for research and practice

To the authors’ knowledge, this is the first survey to assess PA education within a postgraduate curriculum setting. Replication throughout a wider sample of primary care postgraduate deaneries in the UK would help further identify and quantify these findings. The consistency with previous research in undergraduate settings reinforces that these issues still need to be addressed, and highlights the need for GP trainees to gain additional training in the promotion of PA. Although some novel research has been done to address knowledge and experience gaps in undergraduate settings, these need to be extended to postgraduate education, with a particular emphasis on the practical implementation of PA promotion in primary care. Pugh and colleagues have shown in undergraduate settings that self-guided tools can improve knowledge of PA and confidence in giving advice regarding PA,^34^ offering a template for further research in a postgraduate setting. However, concerns regarding PA promotion within medical education have been routinely raised since 2000,^23^ and despite a recurrent acknowledgement of the need for change,^19–22^ progress is not being made.

GPSTs who completed the survey in this study highlighted a limited understanding of UK PA guidelines, a lack of confidence in the implementation of PA promotion, and expressed a lack of education in both their respective undergraduate and postgraduate curriculums. Despite recurrent ‘call to arms’ editorials highlighting the issue, very little original research exists on the topic. This study identifies an urgent need to train the GPs of the future in PA (and effective PA promotion), particularly given the increasing burden of lifestyle-related disease in the UK. A systematic approach within both undergraduate and postgraduate environments is required. A clear theme that emerged from the free-text responses was that present teaching focuses on highlighting the benefits of PA, without providing guidance on how to promote PA effectively. Given the universal acknowledgement of the benefits of PA, postgraduate education could specifically aim to address this issue within primary care. Over the past 20 years, postgraduate medical education has developed from an experience-based to an outcome-based system.^35^ The RCGP has developed specific learning outcomes for GPSTs including knowledge of lifestyle factors, evidence-based approaches, and skills in behavioural change.^36,37^ Assessment ensures enforcement of the curriculum, but while the curriculum includes the above learning outcomes, these are not included in the assessment criteria.^38^

In relation to postgraduate primary care environments, education needs to focus on the practical elements of delivering PA promotion and advice.

## Data

The dataset relied on in this article is available from the corresponding author on reasonable request.
